# Determination of a potential quantitative measure of the state of the lung using lung ultrasound spectroscopy

**DOI:** 10.1038/s41598-017-13078-9

**Published:** 2017-10-06

**Authors:** Libertario Demi, Wim van Hoeve, Ruud J. G. van Sloun, Gino Soldati, Marcello Demi

**Affiliations:** 1 TMC Science and Technology, Brussels, Belgium; 2 Tide Microfluidics, Enschede, The Netherlands; 3 Eindhoven University of Technology, Eindhoven, The Netherlands; 4 Emergency Medicine Unit, Valle del Serchio General Hospital, Lucca, Italy; 5 Medical Image Processing, Fondazione Toscana Gabriele Monasterio, Pisa, Italy

## Abstract

B-lines are ultrasound-imaging artifacts, which correlate with several lung-pathologies. However, their understanding and characterization is still largely incomplete. To further study B-lines, lung-phantoms were developed by trapping a layer of microbubbles in tissue-mimicking gel. To simulate the alveolar size reduction typical of various pathologies, 170 and 80 µm bubbles were used for phantom-type 1 and 2, respectively. A normal alveolar diameter is approximately 280 µm. A LA332 linear-array connected to the ULA-OP platform was used for imaging. Standard ultrasound (US) imaging at 4.5 MHz was performed. Subsequently, a multi-frequency approach was used where images were sequentially generated using orthogonal sub-bands centered at different frequencies (3, 4, 5, and 6 MHz). Results show that B-lines appear predominantly with phantom-type 2. Moreover, the multi-frequency approach revealed that the B-lines originate from a specific portion of the US spectrum. These results can give rise to significant clinical applications since, if further confirmed by extensive *in-vivo* studies, the native frequency of B-lines could provide a quantitative-measure of the state of the lung.

## Introduction

Lung ultrasonography (LUS) was initially thought to be of marginal use because the high mismatch in acoustic impedance between air and soft-tissue causes the lungs to exhibit a very low permeability to ultrasound.

To the contrary, LUS is nowadays a fast-growing field of study since many clinicians acknowledge this diagnostic technique as a powerful, cost-effective, radiation free, and ready available alternative to standard X-ray imaging^1^. However, despite extensive acoustic characterization studies^2–7^ and well documented medical evidence^8–18^, a lot is still unknown about the way US interact with lung tissue.

In fact, LUS still relies on the analysis of imaging artifacts, thus resulting in qualitative and subjective diagnosis, and lacks a dedicated imaging method developed around the acoustical properties of the lungs^8^.

One of the most discussed, and yet not fully understood, lung artifact is the so-called B-line artifact. B-lines (also known as comet tail artifacts) are defined as discrete laser-like vertical hyperechoic reverberation artifacts that arise from the pleural line, extend to the bottom of the screen without fading, and move synchronously with lung sliding. Interestingly, a correlation exists between these artifacts and the increase in extravascular lung water^9,10^, interstitial lung diseases (ILDs)^11,12^, non-cardiogenic lung edema^13^, interstitial pneumonia^14^ and lung contusion^15^.

In this study, in an attempt to better understand the genesis and characteristics of B-lines, we recreated these artifacts in lung-mimicking phantoms. Two different types of phantom were developed, by using two different mono-disperse micro-bubble populations. Microbubbles with a size equal to 170 and 80 µm were used for phantom-type 1 and 2, respectively. This allowed for the analysis of B-lines in a controlled environment where the alveolar size reduction, which is typical of various lung pathologies, was mimicked. Additionally, US images were generated by using an open research platform, thus providing access to the raw data and allowing for the full control of the transmission/reception parameters. These aspects are crucial, since analyzing signals after black/box signal-processing operations would have severely limited the analysis. First, standard B-mode imaging data were acquired, so as to confirm the appearance of B-lines. Subsequently, consecutive narrow-band pulses centered at different frequencies were used for imaging in order to span a large frequency range without employing a single broad-band pulse. This procedure allowed us to investigate the appearance of B-lines as a function of the emitted frequency. In fact, one of the most plausible hypotheses regarding the generation of these artifacts describes B-lines as originating from reverberations between the interstitial spaces, which are enlarged in the presence of specific lung pathologies^8,16^. This will have the effect to locally reduce the acoustic-impedance mismatch with the intercostal tissue and, consequently, to open channels that are accessible to ultrasound. It is thus reasonable to expect the artifact to manifest itself at a specific frequency, which would then correlate to the size and shape of the interstitial space. In such a situation, the native frequency of the B-lines could thus offer a possibility to quantitatively evaluate the state of the lung surface.

## Results

### Visual inspection

**Figure 1:**
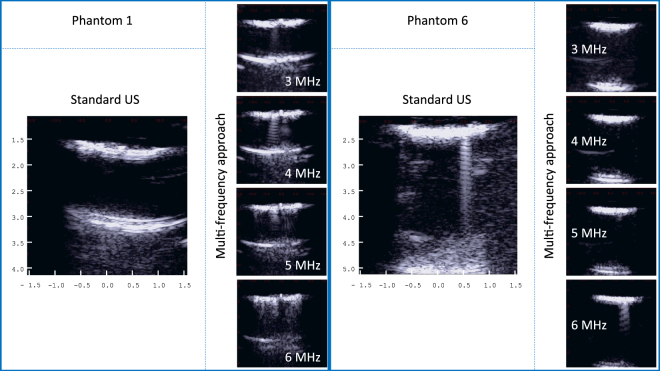
Example of standard US images and multi-frequency US images as obtained from phantom 1 and 6, respectively.

Figure 1 shows an example of standard US images and multi-frequency US images as obtained from phantom number 1 and 6, respectively. As can be seen from the standard US images, a B-line was visible only on phantom 6, which was developed with the smaller microbubbles. Differently, a B-line was visible on both phantoms with the multi-frequency approach. Interestingly, the intensity of the B-line on phantom 1 (obtained with the bigger microbubbles) is stronger at a lower frequency, i.e. 4 MHz, than the B-line on phantom 6, which is shown on the 6 MHz image. The B-line intensity was visually assessed by the sonographer on the ultrasound videos.

It also worth noticing that on the standard US image obtained from phantom 1, A-lines are also visible at a depth of 3 cm. A-lines are horizontal artifacts caused by the reverberation of the ultrasound waves between the transducer and the lung surface (represented here by the bubble cloud positioned at a depth of 1.5 cm, and visible on the US image), and are considered to be an indication of a normal lung surface^1^.

Table 1 reports the results of the visual inspection, which was performed by an experienced sonographer who was unaware of the relation between phantom type and the US video. The phantom type and the number of B-lines observed with both ultrasound imaging modalities are reported in Table 1. As regards the multi-frequency approach, the frequency at which B-lines appeared with the strongest image intensity is also indicated. Overall, considering the total number of B-lines artifacts generated by each phantom type independently on the imaging modality, B-lines appeared predominantly with phantom-type 2 (13 vs 9). Moreover, basing our findings on the multi-frequency approach, it appears that the B-lines generated with phantom-type 2 do appear at a higher frequency (4 to 6 MHz) when compared to the B-lines generated with phantom-type 1 (3 to 5 MHz).

**Table 1:** Results obtained from the visual inspection of the US videos.

Phantom number	Phantom type	Standard US – number of B-lines	Multi-freq. US – number of B-lines	B-lines Frequency [MHz]
1	1	0	3	4
2	1	1	2	5
3	1	0	0	—
4	1	0	3	3
5	1	0	0	—
6	2	2	1	6
7	2	1	1	4
8	2	1	2	5–6
9	2	1	2	5
10	2	1	1	5–6

It is also very important to observe how the appearance of B-lines strongly depends on imaging parameters. This implies that clinicians using different scanners and different probes will generally reach different conclusions, if their findings are simply based on the counting or visual characterization of the artifact.

For the authors, a deeper understanding of the artifact is key for the development of a method which can ultimately provide the clinicians with a more accurate and reliable tool.

### Spectral analysis

**Figure 2:**
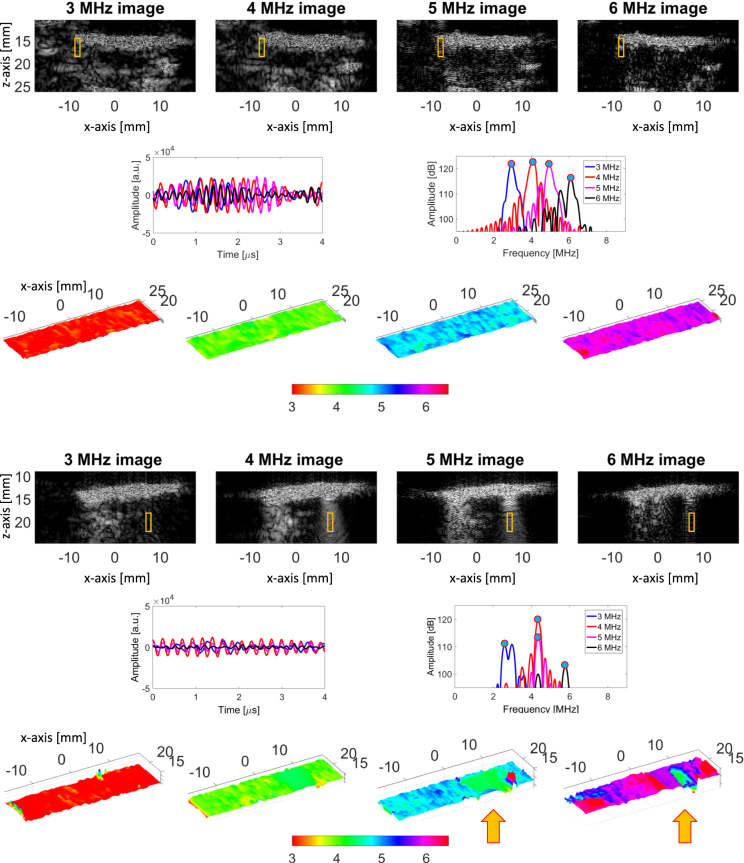
Ultrasound images as obtained from Phantom 5 (top) and 8 (bottom) with the multi-frequency approach are displayed, together with an example of a selected time window (orange box). The time signals and the corresponding frequency profiles are also displayed. Moreover, color maps indicating the estimated frequency of the max of the power spectrum are displayed, together with the corresponding color axis indicating its value in MHz.

Figure 2 shows an example of multi-frequency ultrasound images as obtained from phantom 5 (top) and 8 (bottom). A selected time window (indicated by the orange box), together with the corresponding time signals and frequency profiles, is also displayed. Moreover, color maps indicating the estimated frequency of the maximum magnitude of the power spectrum are shown, together with the corresponding color axis indicating its value in MHz.

In the absence of B-lines, the peak of the power spectra is found to be substantially identical to the center frequency of the ultrasound pulse used to generate each image, as can be seen in the color maps shown for phantom 5.

By contrast, in the presence of a B-line, the peak of the power spectra converges to a specific frequency, which we will refer to as the native frequency of the B-line.

**Figure 3:**
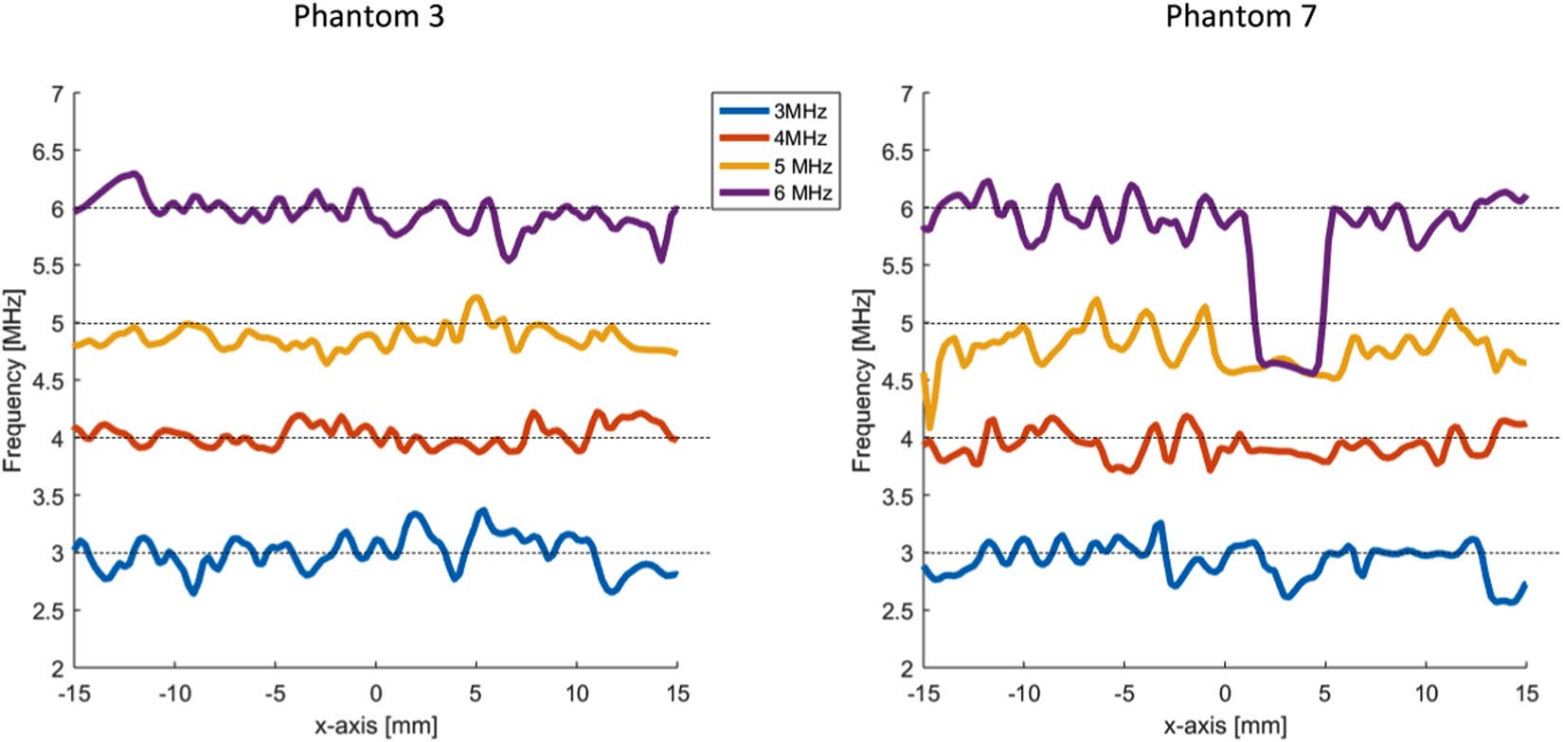
Line graphs displaying a single line of the color maps obtained from phantom 3 and 7.

This phenomenon is further illustrated in Figure 3. Here, line graphs displaying a single line of the color maps obtained from phantom 3 and 7 are shown. As can be seen in Figure 3, the maximum of the power spectrum, which is obtained for phantom 7 when the US pulse centered at 6 MHz is used, shifts towards 4.5 MHz for x ranging from 0 to 5 mm. This corresponds, in the ultrasound images, to the location of a B-line.

## Discussion and Conclusion

In this paper, we have presented results from an *in-vitro* study aimed at deepening our understanding of B-line artifacts.

Two lung phantom types designed to mimic the alveolar size reduction, which is typical of several lung pathologies, were imaged using an open research platform.

These phantoms were able to generate B-line artifacts.

Standard ultrasound imaging and a multi-frequency imaging approach were used. Results show that, when standard imaging is adopted, B-lines are substantially only visible on phantom 2, which is the phantom with the smaller bubbles. This suggests a relation between bubble size (the air-space size) reduction and increased artifact formation. This observation is in line with the correlation between B-lines and a pathological condition of the lung.

When the multi frequency approach is adopted, more B-lines are visible (15 vs 7). Moreover, this imaging modality indicates that B-line formation is strongly frequency dependent, and generally dependent on the imaging modality.

Additionally, the multi-frequency approach allows us to observe that the B-lines obtained from phantom 2 are generated at higher frequencies (4 to 6 MHz) when compared to those emerging from phantom 1 (3 to 5 MHz). In particular, B-lines appeared with significantly stronger amplitude in one of the four images obtained with the multi-frequency approach, and spectral-analysis confirmed that B-lines originate from a specific portion of the US spectrum. This means, on the one hand that the native frequency of the B-lines may be used to quantitatively evaluate the state of the lung surface, while on the other it shows that there is a need for the development of an ultrasound method that is specific and dedicated to the lung if we want to achieve reproducible and reliable medical findings by means of LUS.

Another recent attempt in this direction is the work done by K.Mohanty *et al*.^19^. Differently than the approach we proposed, in this paper the authors investigated (*ex-vivo* and *in-vivo*) the possibility to use diffusion constant and transport mean free path estimation as a way to quantitatively characterize the micro-architecture of the lung parenchyma, and ultimately predict the extent of pulmonary edema. Their results showed how methods based on ultrasound multiple scattering could provide important information for the quantitative characterization of the lung parenchyma.

We hope that research attention in this direction will grow, and help to increase the precision and acceptance of lung ultrasonography.

To this end, our future work will focus on the validation of the results presented in this study by means of an extensive clinical study. A key aspect will be to investigate the link between the native frequency of B-lines and the condition of the lung. In parallel, new *in-vitro* study will be performed in order to assess how accurately we can relate the native frequency of the B-lines to the size and distribution of the air spaces.

## Methods

### Lung mimicking phantoms

Two monodisperse microbubble populations with different diameters were generated by using the MicroSphere Creator® (Tide Microfluidics, Enschede, the Netherlands). These microbubbles consisted of air-bubbles encapsulated in a lipid shell. Subsequently, ten lung-mimicking phantoms were developed. These consisted of a layer of one of the above-mentioned microbubble populations trapped in tissue-mimicking gel. The microbubbles had a diameter equal to 170 and 80 µm, and were used to develop five type 1 phantoms and five type 2 phantoms, respectively. The biggest bubble size was defined by the largest bubbles producible by the MicroSphere Creator®. Next, the smallest bubble size was defined in order to achieve a good separation between the two bubbles population.

Both sizes were also chosen to attain a cluster of air bubbles which were smaller in size than a normal alveolar sac, whose diameter is approximately 280 µm^20^.

**Figure 4:**
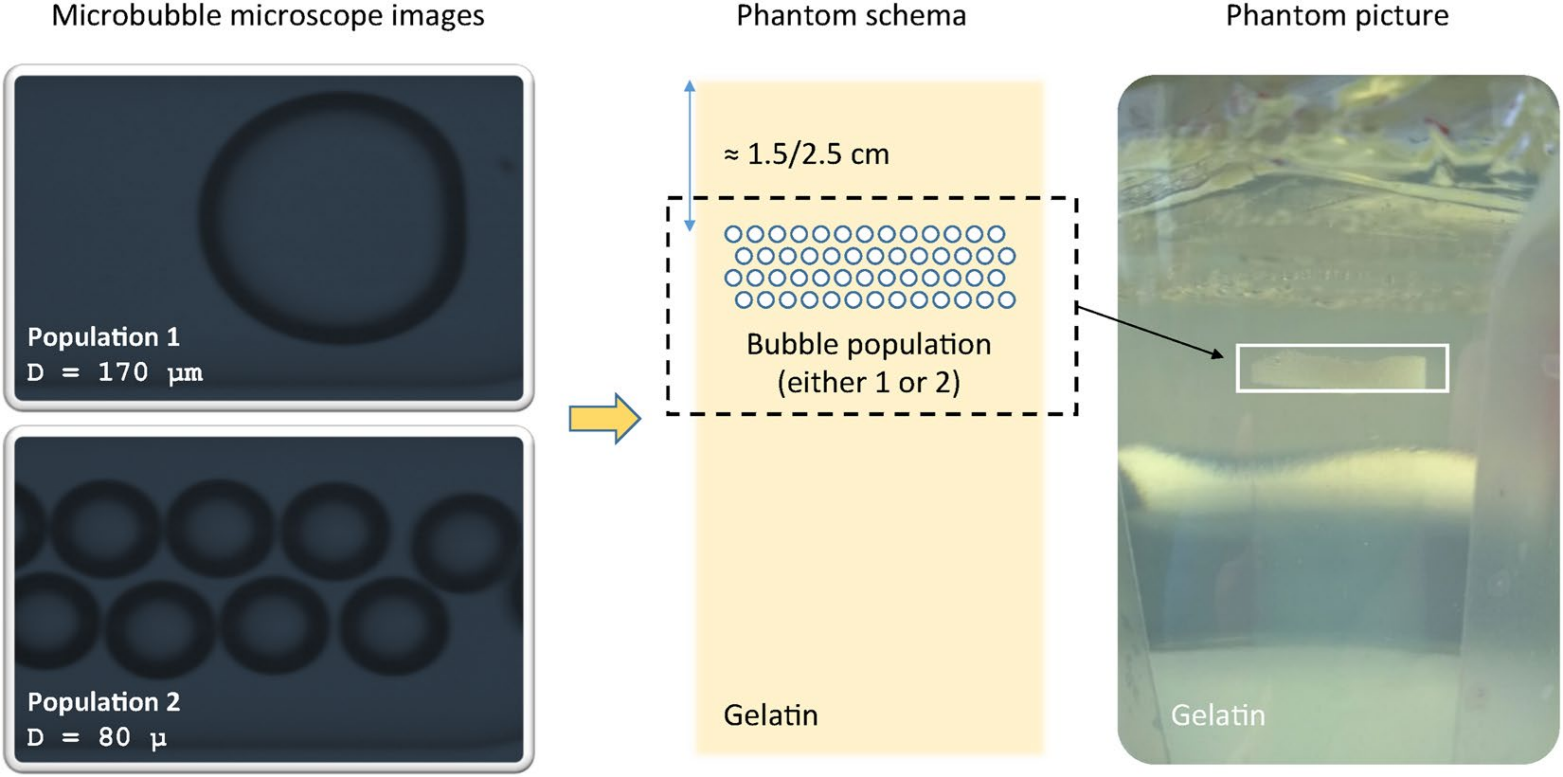
Microscope images of the generated microbubbles, together with a phantom schema and a picture of a lung mimicking phantom.

Microscope images of the generated bubbles are shown in Fig. 4, together with a phantom schema and a picture of a lung mimicking phantom. The clouds of microbubbles were positioned at a depth of approximately 1.5 to 2.5 cm from the top of the gelatin phantom, where the US probe was positioned for imaging.

In the literature, alternative experimental models able to generate B-lines have been reported. Differently than the model presented in this study these models were based on gelatin phantoms containing large air bubbles (mm size) obtained by manual injection of air by means of a syringe^21^, sponges^22,23^ and foams^24^.

### Imaging protocol

A LA332® (Esaote, Florence, Italy) linear-array probe was connected to the ULA-OP research platform^25^ and used for imaging. A sub-aperture consisting of 64 elements was used in transmit and receive mode, and was linearly shifted over the array to obtain a final image consisting of 129 lines. In transmit mode, the focus was set at the depth of the microbubble cloud, and dynamic beamforming was used in receive mode. Time gain compensation parameters were set experimentally to obtain a good signal to noise ratio (and to avoid saturation) for the signals that were measured at greater depths than the bubble cloud, and were maintained unmodified for all the acquisitions.

Here, with the term “greater depths” we intend received after a longer time delay.

First, standard US imaging at 4.5 MHz was performed. In this case a 0.5-µs Gaussian pulse was used as a driving signal.

**Figure 5:**
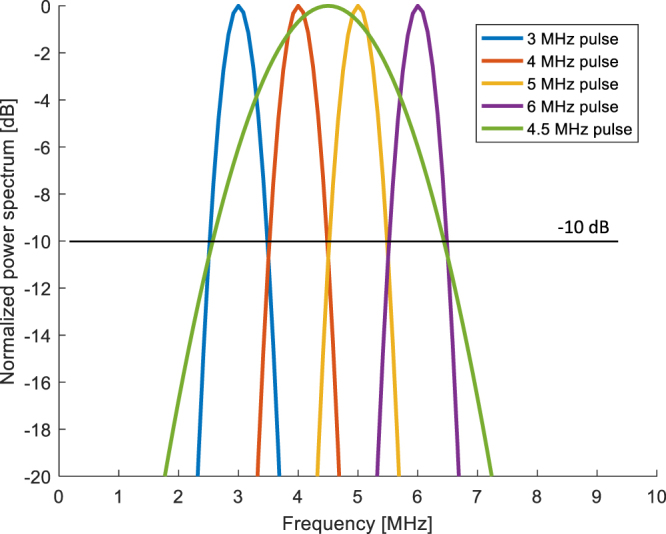
Normalized frequency spectra of the driving signals. The 4.5 MHz pulse was used to perform standard US imaging, while the other pulses were used for the multi-frequency approach.

Subsequently, a multi-frequency approach was used: images were sequentially generated using orthogonal sub-bands centered at different center frequencies, i.e., at 3, 4, 5, and 6 MHz. To keep the absolute bandwidth constant when transmitting at the different center frequencies, an absolute pulse time-length of 2 µs was maintained for every frequency. Moreover, in this way the different pulses allowed us to cover the same bandwidth excited by the 4.5 MHz pulse, and guarantee a −10 dB orthogonality in the frequency domain. Figure 5 shows the normalized power spectra of the different driving signals. The echo signals received at the probe were digitized at a sampling frequency of 50 MHz.

For each phantom, standard US imaging at 4.5 MHz was performed first. The US probe was manually operated and translated so as to cover the entire bubble cloud. An US video was thus recorded.

Subsequently, the number of B-lines was visually assessed by an expert sonographer who was unaware of the phantom type that corresponded to each US video. The same procedure was then repeated with the multi-frequency approach, where the four images corresponding to the four different frequencies were displayed in real time. Subsequently, the probe was positioned back at the location where a B-line was observed, and raw data were recorded with the multi-frequency modality.

The data necessary to form ten image-frames were recorded for each transmitted frequency.

### Raw data analysis

**Figure 6:**
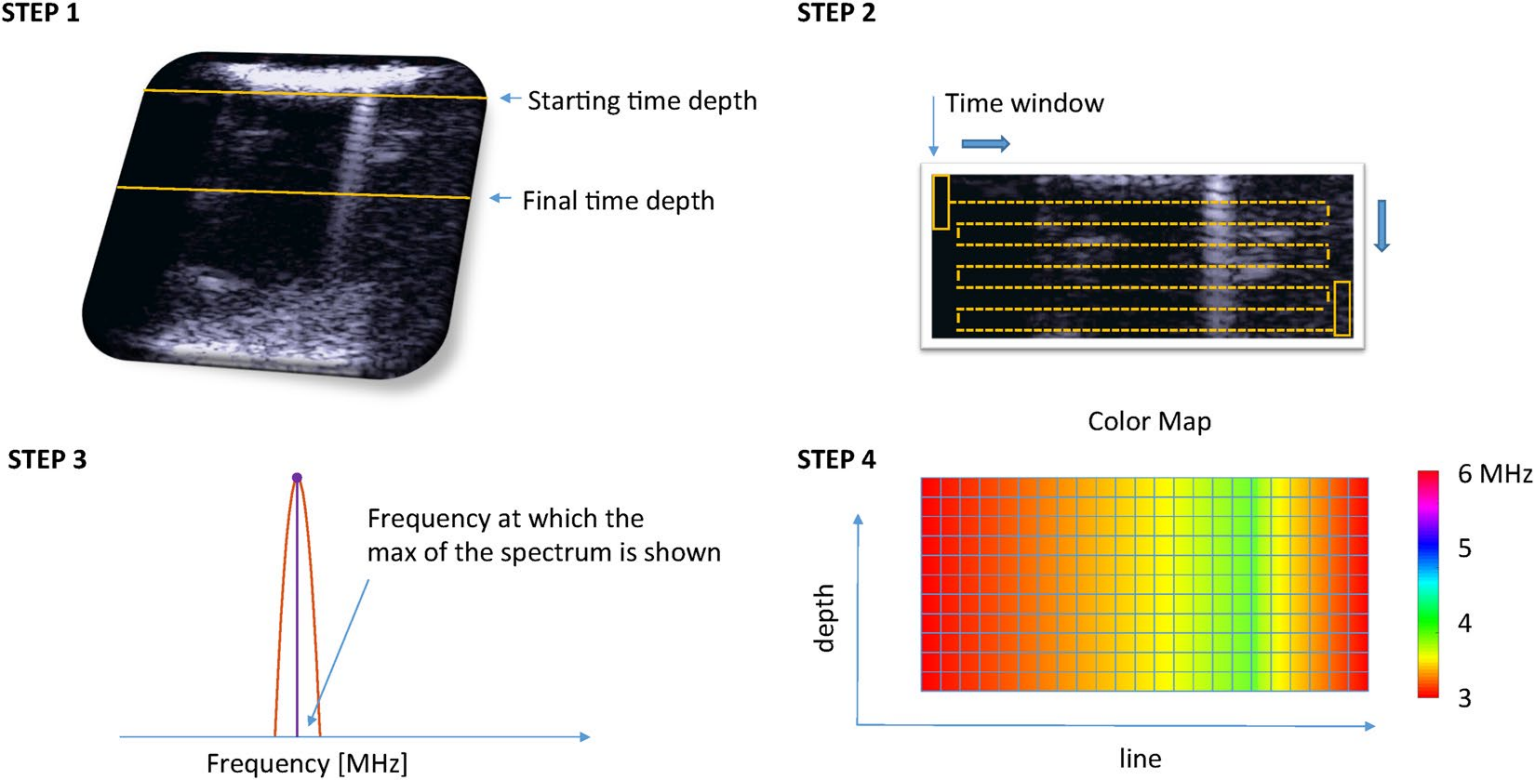
Schematic representation of the method that was used to process the raw data.

The US raw data were processed to analyze the spectra of the signals received from the phantoms. A four-step process was applied. In step 1, a part of the data which started from the time depth at which the first echoes (just after the bubble cloud) were received was selected. This, when compared to *in-vivo* data, is equivalent to analyzing the ultrasound echoes received after the pleura line^1^. The final time depth to be selected was then obtained by considering a time depth up to a maximum of 13 µs (approximately 1 cm) from the starting time depth. In step 2, a time window of 4 µs was extracted from the selected data. This time window was then shifted by one line (horizontally) and by a 0.02 µs time-step (vertically) so as to cover the entire data-set. This process resulted in 129 time signals for each of the (650) selected depths for each (10) frame of each multi-frequency approach (4) image as obtained from each (10) phantom. These signals were FFT transformed in step 3, and their power spectra analyzed. In particular, the location of the peak in the power spectra was evaluated. In step 4, a 129 (lines) by 650 (depths) by 10 (frames) matrix with values ranging from 3 to 6 MHz was obtained for each transmitted frequency. This matrix was averaged over the frames, and a color map was obtained for each image generated with the multi-frequency approach. Figure 6 visually describes the procedure. Apart from the selection of the starting time-depth, the process was automated and implemented in Matlab®.

## Electronic supplementary material

US Video - Phantom 1

US Video - Phantom 6
